# Thymoquinone-Loaded Nanostructured Lipid Carrier Exhibited Cytotoxicity towards Breast Cancer Cell Lines (MDA-MB-231 and MCF-7) and Cervical Cancer Cell Lines (HeLa and SiHa)

**DOI:** 10.1155/2015/263131

**Published:** 2015-01-06

**Authors:** Wei Keat Ng, Latifah Saiful Yazan, Li Hua Yap, Wan Abd Ghani Wan Nor Hafiza, Chee Wun How, Rasedee Abdullah

**Affiliations:** ^1^Laboratory of Molecular Biomedicine, Institute of Bioscience, Universiti Putra Malaysia (UPM), 43400 Serdang, Selangor, Malaysia; ^2^Department of Biomedical Science, Faculty of Medicine and Health Sciences, Universiti Putra Malaysia (UPM), 43400 Serdang, Selangor, Malaysia; ^3^Laboratory of Vaccine and Immunotherapeutics, Institute of Bioscience, Universiti Putra Malaysia (UPM), 43400 Serdang, Selangor, Malaysia; ^4^Department of Veterinary Pathology and Microbiology, Faculty of Veterinary Medicine, Universiti Putra Malaysia (UPM), 43400 Serdang, Selangor, Malaysia

## Abstract

Thymoquinone (TQ) has been shown to exhibit antitumor properties. Thymoquinone-loaded nanostructured lipid carrier (TQ-NLC) was developed to improve the bioavailability and cytotoxicity of TQ. This study was conducted to determine the cytotoxic effects of TQ-NLC on breast cancer (MDA-MB-231 and MCF-7) and cervical cancer cell lines (HeLa and SiHa). TQ-NLC was prepared by applying the hot high pressure homogenization technique. The mean particle size of TQ-NLC was 35.66 ± 0.1235 nm with a narrow polydispersity index (PDI) lower than 0.25. The zeta potential of TQ-NLC was greater than −30 mV. Polysorbate 80 helps to increase the stability of TQ-NLC. Differential scanning calorimetry showed that TQ-NLC has a melting point of 56.73°C, which is lower than that of the bulk material. The encapsulation efficiency of TQ in TQ-NLC was 97.63 ± 0.1798% as determined by HPLC analysis. TQ-NLC exhibited antiproliferative activity towards all the cell lines in a dose-dependent manner which was most cytotoxic towards MDA-MB-231 cells. Cell shrinkage was noted following treatment of MDA-MB-231 cells with TQ-NLC with an increase of apoptotic cell population (*P* < 0.05). TQ-NLC also induced cell cycle arrest. TQ-NLC was most cytotoxic towards MDA-MB-231 cells. It induced apoptosis and cell cycle arrest in the cells.

## 1. Introduction

Cancer is one of the major causes of death in the world [1]. Breast cancer and cervical cancer are the two most common malignancies among women worldwide. It is estimated that over 1.3 million new cases of breast cancer are diagnosed every year globally, of which over 450,000 of the patients would die from the disease. Although the cervical cancer incidence and mortality rate have declined, more than 520,000 cervical cancer new cases and over 275,000 deaths have been reported in 2008 worldwide [2].

*Nigella sativa* (also known as black seed or* habbatus sauda*) appears as one of the important herbs among various medicinal plants. Majority of the biological activities of* Nigella sativa* are associated with the presence of thymoquinone (TQ), the major bioactive compound found in the seeds of the plant [3]. TQ or 2-isopropyl-5-methyl-1,4-benzoquinone (C_10_H_12_O_2_) with relative molecular mass of 164.2 exhibited strong cytotoxic activities against several cancer cell lines including human cervical adenocarcinoma (HeLa) [4], human squamous carcinoma (SiHa) [5], human oestrogen receptor negative breast adenocarcinoma (MDA-MB-231), and human oestrogen receptor positive breast adenocarcinoma (MCF-7) [6, 7]. Intraperitoneal route has been used to administer TQ. Nevertheless, this route of administration in preclinical and clinical use is restricted by high discomfort and costly and sterility issues. Although oral delivery of TQ is valuable, it is limited by the solubility-related poor oral bioavailability [8]. The solubility of pure TQ is relatively low in water [9].

In order to overcome the low solubility and bioavailability of the active compounds, colloidal drug carrier systems such as nanostructured lipid carriers (NLCs) have been developed as drug delivery vehicles [10]. By having a mixture of solid and liquid lipids, NLC serves as a good drug delivery vehicle. It provides many advantages including capability of increasing the bioavailability of poorly soluble compounds, providing protection for sensitive active compounds, and facilitating controlled release of drugs [11, 12].

In the present study, thymoquinone-loaded nanostructured lipid carrier (TQ-NLC) was formulated. The physicochemical characteristics and stability of TQ-NLC were evaluated, and* in vitro *cytotoxicity towards breast cancer cell lines (MCF-7 and MDA-MB-231) and cervical cancer cell lines (HeLa and SiHa) was determined. The mode of cell death and cell cycle arrest induced by TQ-NLC in MDA-MB-231 cells were also evaluated.

## 2. Materials and Methods

### 2.1. Reagents

Hydrogenated palm oil (Softisan 154) was obtained from Condea (Witten, Germany). Olive oil (Basso) was obtained from Basso Fegele and Figli Srl (San Michele Di Serino, Italy). Eagle's minimal essential medium (EMEM), thymoquinone (TQ), 3-(4,5-dimethylthiazol-2-yl)-2,5-diphenyltetrazolium bromide (MTT) powder, trypan blue dye solution, propidium iodide (PI), thimerosal, and sorbitol were purchased from Sigma-Aldrich (St. Louis, USA). RPMI-1640 tissue culture medium, penicillin/streptomycin antibiotic, Mycoplex foetal bovine serum (FBS), and trypsin-EDTA were purchased from PAA Laboratories (Linz, Austria). Other reagents used were lecithin, a form of phospholipid (Cologne, Germany), nonionic surfactant Polysorbate 80 (Fisher-Scientific, USA), and HPLC grade methanol (Merck, USA).

### 2.2. Preparation of Lipid Matrices

The lipid matrices were prepared as previously described [13]. Briefly, hydrogenated palm oil, lecithin, and olive oil were mixed. The mixture was heated to 70°C (approximately 10°C above the melting point of the lipid matrices). After stirring with a teflon-coated magnet, a yellowish-milky solution was obtained.

### 2.3. Preparation of Aqueous Surfactant Matrices

The aqueous surfactant mixture was prepared as previously described [13]. Briefly, sorbitol, nonionic surfactant (Polysorbate 80), and thimerosal were dissolved in deionized water (18.2 MΩ·cm). The solution was heated to 70°C (same temperature as the TQ-loaded lipid matrices).

### 2.4. Synthesis of Blank NLC and TQ-NLC

Prior to dispersion into the aqueous surfactant mixture, TQ was added into the lipid matrix. At 70°C, 5% of TQ-loaded lipid matrices were dispersed into the aqueous surfactant mixture with high-speed stirring by the Ultra-Turrax (IKA, Staufen, Germany) at 13,000 rpm for 10 minutes to produce a hot preemulsion. The hot preemulsions were homogenized using a high-pressure homogenizer EmulsiFlex (Avestin, Inc., Ottawa, Canada) at 500 bars for 40 cycles. The emulsions were allowed to recrystallize at room temperature for 24 hours to form TQ-NLC [13]. Blank NLC was also synthesized without addition of TQ into the lipid matrix.

### 2.5. Measurement of the Particle Size and Polydispersity Index (PDI)

The average diameter and polydispersity index (PDI) of TQ-NLC were analyzed at a fixed angle of 173° and at 25°C with the Malvern software using photon correlation spectroscopy (PCS) (Zetasizer Nano ZS, Malvern, UK). TQ-NLC was diluted with deionized water (1 : 9) prior to analysis to prevent back-scattering effect. The analysis was performed in triplicate [11].

### 2.6. Measurement of the Zeta Potential

The electrostatic charge on the surface of TQ-NLC was analyzed by using a laser Doppler electrophoresis technique, performed by Zetasizer Nano ZS (Malvern, UK) at pH 5.2. The results were expressed as zeta potential. TQ-NLC was diluted with deionized water (1 : 9) prior to analysis. The analysis was performed in triplicate [11].

### 2.7. Determination of TQ-NLC Encapsulation Efficiency and Drug Loading Capacity

Encapsulation efficiency (EE) and drug loading capacity of TQ-NLC were calculated by determining the amount of free drug using an ultrafiltration technique. Briefly, 5 mL of TQ-NLC solution was placed in the upper chamber of a centrifuge tube matched with an ultrafilter (Amicon Ultra, Millipore Co., USA, MWCO 10 kDa) and centrifuged for 10 minutes at 2000 ×g. The ultrafiltrate containing the unencapsulated drug was determined by high performance liquid chromatography (HPLC) analysis. The drug loading content was the ratio of incorporated drug to lipid (w/w). The TQ encapsulation efficiency and drug loading capacity were calculated by the following equation:

(1) $$\begin{array}{c} \text{TQ}\;\;\text{encapsulation}\;\;\text{efficiency}=\frac{W_{\text{total}\;\;\text{drug}}-W_{\text{free}\;\;\text{drug}}}{W_{\text{total}\;\;\text{drug}}}\times 100,\\ \text{Drug}\;\;\text{loading}\;\;\text{capacity}=\frac{W_{\text{total}\;\;\text{drug}}-W_{\text{free}\;\;\text{drug}}}{W_{\text{lipid}}}\times 100, \end{array}$$

where “*W*_total  drug_” is the mass of the total TQ used, “*W*_free  drug_” is the mass of the free drug detected in the filtrate of lower chamber of postcentrifugation of the aqueous dispersion, and “*W*_lipid_” is the mass of lipid added into the aqueous matrix [14].

### 2.8. HPLC Analysis of Free TQ

HPLC analysis was performed by a Waters Alliance HPLC System (Milford, MA, USA) equipped with a photodiode array detector. The stationary phase comprised of a Merck HSS-T-3 C18 (100 × 2.1 mm, 1.8 mm) HPLC column was maintained at 30°C. The mobile phase consisted of a mixture of methanol (75%) and water (25%), which was pumped at a flow rate of 1.0 mL/min. The injection volume was 10 *μ*L and analysis was performed at 255 nm wavelength with a total run time of 5 min. Data acquisition, data handling, and instrument control were performed by Empower Software v1.0. (Milford, MA, USA).

### 2.9. Stability Test

The formulations (NLC and TQ-NLC) were stored at room temperature (25°C) for 6 months. Subsequently, the average diameter, polydispersity index (PDI), zeta potential, and encapsulation efficiency of TQ-NLC were again evaluated.

### 2.10. Differential Scanning Calorimetry (DSC)

Differential scanning calorimetry (DSC) analysis was done with Mettler DSC 822e (Mettler Toledo, Greifensee, Switzerland). Prior to analysis, TQ-NLC dispersion was freeze-dried. Approximately 10 mg of bulk lipid, free NLC, TQ, and TQ-NLC were placed in aluminium pans. The pan was heated and the thermograms were recorded at temperature range of 25 to 70°C at a heating rate of 5°C/min.

### 2.11. Cell Culture

The human breast adenocarcinoma (MCF-7 and MDA-MB-231), human cervical adenocarcinoma (HeLa), human cervical squamous cell carcinoma (SiHa), Swiss mouse embryo fibroblast (3T3-L1), and African green monkey kidney epithelial (Vero) cell lines were purchased from the American Type and Culture Collection (ATCC) (Rockville, MD, USA). SiHa cells were grown in EMEM, while MCF-7, MDA-MB-231, HeLa, 3T3-L1, and Vero cells were maintained in RPMI-1640. Both media were supplemented with 10% FBS and 1% antibiotics (100 IU/mL penicillin and 100 *μ*g/mL streptomycin). The cells were maintained at 37°C in a humidified atmosphere of 5% CO_2_.

### 2.12. Determination of Cytotoxicity of TQ-NLC

The cells were treated with various concentrations of TQ-NLC (3.125 to 100 *μ*M) in a 96-well plate for 24, 48, and 72 hours. Control was also included. MTT solution (5 mg/mL) was added and the plate was incubated for 3 hours. DMSO was then added to dissolve the dark-blue formazan crystals. The absorbance at 570 nm and the reference wavelength of 630 nm were measured with a microplate reader (Opsys MR, USA) [15]. Cell viability was calculated by the following formula:

(2) $$\begin{array}{c} \text{Cell}\;\;\text{viability}=\left[\frac{\left({\text{OD}}_{\text{treated}}-{\text{OD}}_{\text{blank}}\right)}{\left({\text{OD}}_{\text{control}}-{\text{OD}}_{\text{blank}}\right)}\right]\times 100\%. \end{array}$$

### 2.13. Morphological Analysis

MDA-MB-231 cells were treated with TQ-NLC at concentration of 3.125 and 6.25 *μ*M for 24, 48, and 72 hours. Control (untreated cells) was also included. The changes in cell morphology were examined under an inverted light microscope (Olympus, Tokyo, Japan).

### 2.14. Cell Cycle Analysis

MDA-MB-231 cells were treated with TQ-NLC at concentration of 3.125 and 6.25 *μ*M for 24 and 48 hours. Control (untreated cells) was also included. Following treatment, the cells were harvested by trypsinization, followed by centrifugation at 300 ×g for 10 minutes. The supernatant was discarded and the pellet was washed twice with ice-cold PBS. Cell pellets were resuspended vigorously and fixed with 70% ethanol and kept at −20°C for 2 hours. The cells were then centrifuged at 300 ×g for 10 minutes at 4°C, and ethanol was discarded. The cells were washed with ice-cold PBS and centrifuged again at 200 ×g for 10 minutes at 4°C. The pellets were resuspended in a solution containing 425 *μ*L of PBS, 50 *μ*L of RNase A (1 mg/mL), and 25 *μ*L of propidium iodide (1 mg/mL) and incubated for 15 minutes at 4°C before analysis by a flow cytometer (FACSCalibur, BD Biosciences, USA). The population of cells in each cell-cycle phase was determined by CellQuest Pro Software (BD Bioscience, USA) [5]. The population of cells in each phase was determined by using ModFit LT (Verity Software House).

### 2.15. Statistical Analysis

Data were analyzed with one-way analysis of variance (ANOVA) and Duncan's multiple range test (DMRT) using Statistical Package for Social Science (SPSS) version 21.0. All the data were expressed as mean ± standard error of mean (SEM) and *P* < 0.05 was considered significant.

## 3. Results

### 3.1. Physicochemical Characteristic of NLC and TQ-NLC

**Figure 1:**
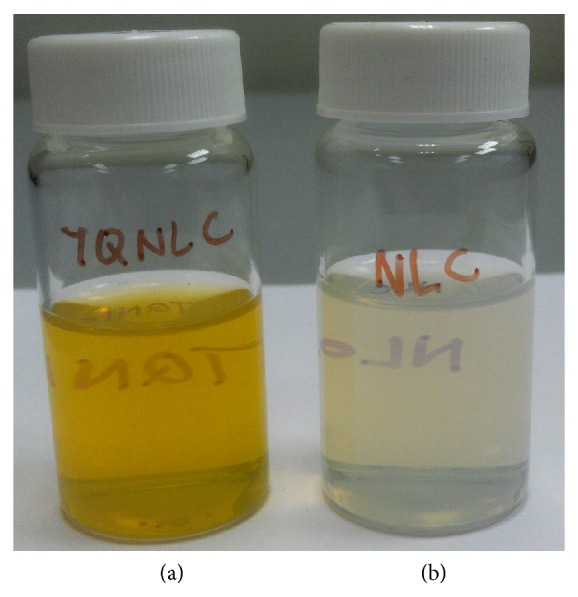
(a) TQ-NLC and (b) blank NLC 24 hours after synthesis.

Following preparation, 100 mL of blank nanostructured lipid carriers (NLCs) and thymoquinone-loaded nanostructured lipid carriers (TQ-NLCs) was synthesized. TQ-NLC and NLC presented as a bright yellowish opalescent and milky whitish dispersion, respectively (Figure 1).

The physicochemical characteristics of NLC and TQ-NLC are shown in Table 1. Both formulations show average diameter less than 50 nm, polydispersity index (PDI) below 0.25, and negative zeta potential, regardless of the duration of storage.

**Figure 2:**
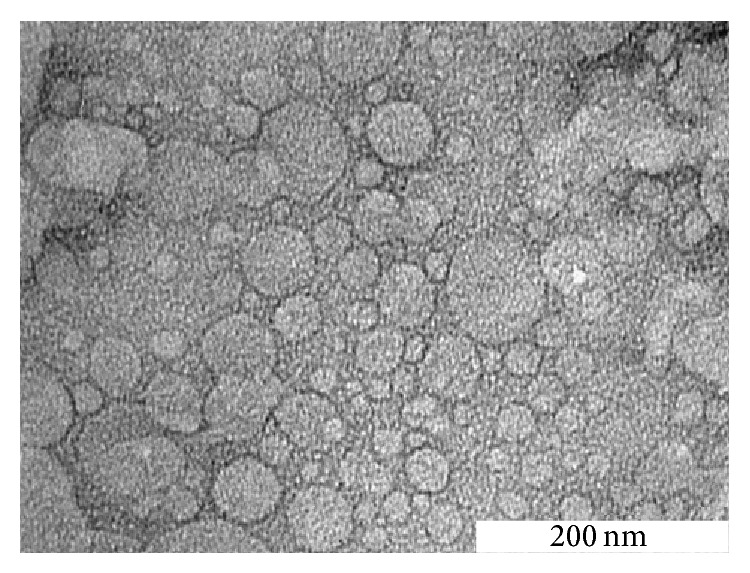
Transmission electron micrograph of TQ-NLC after 24 hours of recrystallization (magnification 150000x).

Figure 2 shows the transmission electron micrograph of the TQ-NLC. TQ-NLC appeared spherical with dark grey shading. No TQ crystals were detected in the micrograph. The micrograph reveals that majority of TQ-NLC has the diameter less than 50 nm.

### 3.2. TQ-NLC Encapsulation Efficiency and Drug Loading Capacity

Following ultrafiltration, concentration of free TQ was analyzed by using HPLC, and the drug encapsulation efficiency as well as drug loading capacity was calculated. The drug encapsulation efficiency of TQ-NLC stored for 0 weeks (24 hours after synthesis) and 24 weeks after synthesis was significantly different (*P* < 0.05) (Table 2).

### 3.3. Differential Scanning Calorimetry (DSC)

**Figure 3:**
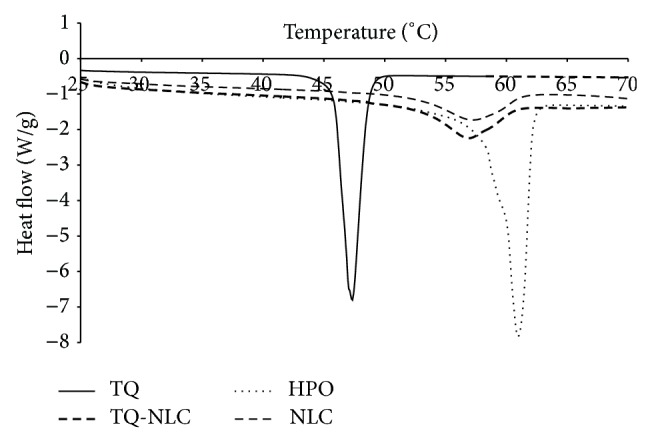
Thermogram recorded as a function of temperature from 25 to 70°C.

The melting point of hydrogenated palm oil (HPO), NLC, TQ, and TQ-NLC determined by using Mettler DSC 822e machine was 60.50, 57.18, 47.35, and 56.73°C, respectively (Figure 3).

### 3.4. Cytotoxicity of TQ-NLC

The percentage of cell viability of MCF-7, MDA-MB-231, HeLa, SiHa, 3T3-L1, and Vero treated TQ-NLC reduced significantly (*P* < 0.05) at all the studied concentrations as compared to control. Among the cancerous cell lines, the IC50 values determined from the MTT assay indicated that TQ-NLC was most potent towards MDA-MB-231, followed by SiHa, HeLa, and MCF-7. Nevertheless, TQ-NLC was found to be relatively nontoxic towards normal cell lines (3T3-L1 and Vero) at 72 hours of incubation time (Table 3). Based on this, further analysis was carried out only on MDA-MB-231 cells.

In order to show that the cytotoxicity of TQ-NLC was due to the active compound, TQ, the cells were treated with the highest corresponding concentration of blank NLC (0.32%) for 24, 48, and 72 hours. The percentage of cell viability was more than 87%.

### 3.5. Morphological Changes of MDA-MB-231 Cells Treated with TQ-NLC

**Figure 4:**
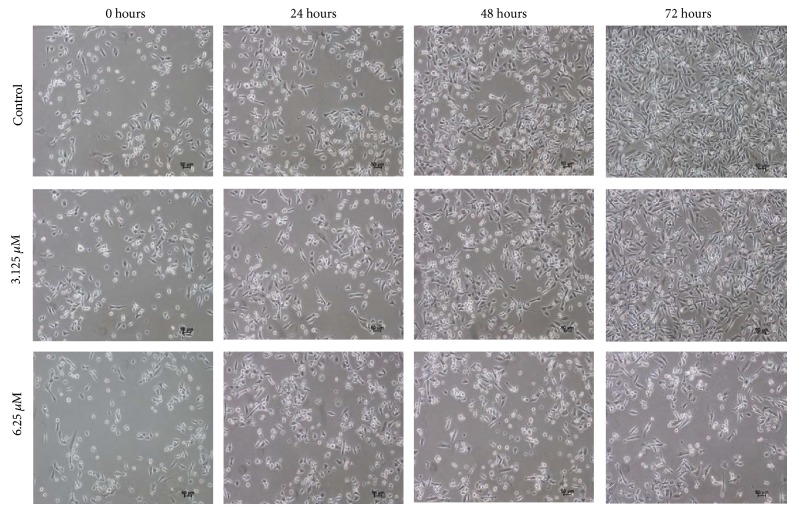
Morphological changes of MDA-MB-231 cells treated with TQ-NLC observed under an inverted light microscope. Control untreated cells were also included (100x magnification).

As shown in Figure 4, TQ-NLC caused morphological changes in MDA-MB-231 cells. Reduction in cells number was obvious at higher concentration of TQ-NLC (6.25 *μ*M). Majority of the cells detached from the substratum as early as at 24 hours.

### 3.6. Effects of TQ-NLC on the Cell Cycle of MDA-MB-231 Cells

**Figure 5:**
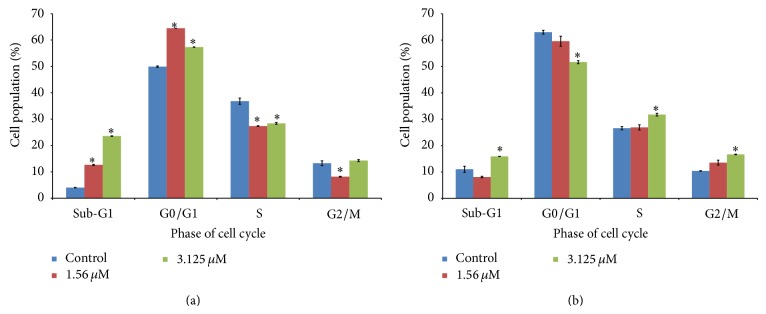
Cell cycle analysis of MDA-MB-231 cells treated with TQ-NLC for (a) 24 hours and (b) 48 hours. Data are presented as mean ± SEM of duplicate samples. ∗ indicates significant difference from untreated control group (*P* < 0.05).

**Figure 6:**
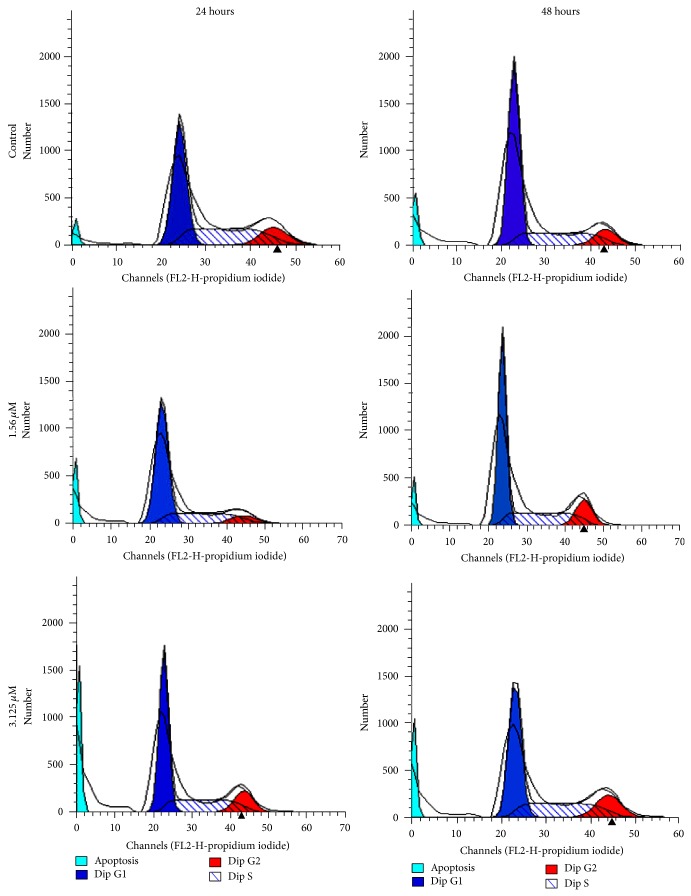
Cell cycle analysis of MDA-MB-231 cells treated with TQ-NLC for 24 hours and 48 hours, performed by flow cytometer. An increase in the cell population at sub-G1 phase was noted after treatment with 1.56 and 3.125 μM of TQ-NLC.

An increase in the cell population at sub-G1 phase was noted after treatment with 1.56 and 3.125 *μ*M of TQ-NLC (*P* < 0.05). Increase in G2/M and S phase population was noted at 48 hours (*P* < 0.05) (Figures 5 and 6).

## 4. Discussion

The synthesis of TQ-NLC involves three main major processes that include lipid and aqueous matrices formulation, high-speed stirring by the Ultra-Turrax, and homogenization by the high-pressure homogenizer EmulsiFlex. Solid and liquid lipids were utilized to provide a core composed of highly lipophilic environment to accommodate TQ, thus becoming a suitable and optimum nanocarrier or reservoir for the compound. The incorporation of solid and liquid lipid mixture in the lipid matrix promotes less perfect crystallization, thus lowering the probability of encapsulated drug expulsion upon storage. Besides, TQ as a lipophilic active compound has greater solubility in liquid lipids than that of solid lipids, which allows more flexibility for modulation of drug release and better drug-loading efficiency [16].

It is known that soft nanoscale particle that includes lipid nanoparticles and NLC is less feasible in achieving particle size of less than 100 nm as compared to the hard material such as metal oxide [17]. Nevertheless, TQ-NLC with the average diameter of 35.66 ± 0.1235 nm (submicron size) has been successfully synthesized in our study. This was proven by the formation of larger nanoparticles after introduction of TQ to the system compared to blank NLC. A nanoscale particle like TQ-NLC, with the particle diameter less than 100 nm, exhibits unique physical and biological properties, making it particularly ideal for drug encapsulation, and provides a large surface area for the reaction with its target components [17]. Furthermore, nanoscale size minimizes the probability of TQ-NLC being phagocytosed by macrophage of mononuclear phagocytic system, hence decreasing the destruction of TQ-NLC in the body [18]. With that, it is postulated that the biological activities of TQ will be retained and will be able to be fully utilized by the targeted system.

PDI is a measurement of particle homogeneity that varies from 0 to 1. The polydispersity index (PDI) of TQ-NLC of 0.177 ± 0.0024 indicates that all the nanostructured particles of TQ-NLC were almost in monodispersity and homogeneous with narrow size distribution. The closer the value of PDI to zero, the higher the homology between the particles [19]. The PDI of less than 0.5 also suggests that there was no aggregation of the nanoparticle of TQ-NLC as PDI more than 0.5 is an indication of particle aggregation [20]. The aggregates do not interact with living organisms in the way smaller individual particles do. The aggregation or agglomeration impedes the targeting efficiency of nanoscale particle to cells and tissues. In addition, the degree of cellular uptake and cytotoxicity might be reduced due to the presence of unwanted aggregates since aggregation increases the particle size and lowers the surface area. Unwanted aggregates may settle out of suspension and be no longer bioavailable [21–23].

Zeta potential has been touted as one of the paramount factors for evaluating the stability of colloids. The zeta potential value of TQ-NLC was −16.72 ± 0.4474 mV. Zeta potential referred to the electrostatic charges on the surface of the nanoparticles in the suspension, which can be used to predict the long term stability of the nanoparticles [11]. Since the zeta potentials above 30 mV or below −30 mV were required for full electrostatic stabilization [24], electrostatic charges on the surface of TQ-NLC can be considered not able to keep the formulation stable during the investigated period (6 months). Nevertheless, in our studies, TQ-NLC was found stable up to 24 weeks (6 months) as the average diameter remained lower than 100 nm although there was a significant increase from 35.66 ± 0.1235 nm to 37.05 ± 0.2742 (*P* < 0.05). Many experiments demonstrated that it is not only electrostatic repulsion dominates the stability of any nanoparticles, but also the use of steric stabilizer that favour the formation of stable nanoparticle dispersion. High surfactant mixture can easily compensate missing electrostatic repulsion to stabilize the dispersion for long time. Hence, Polysorbate 80 was used in the production of TQ-NLC as a stabilizer in the aqueous matrix to maintain the stability. Furthermore, the steric hindrance from Polysorbate 80 has an additional effect in increasing the particle stability [24, 25]. The addition of Polysorbate 80 in the synthesis of TQ-NLC which aims to increase the stability and reduce aggregation was confirmed by the low polydispersity index of TQ-NLC. It was suggested that concentration of 1.5% of Polysorbate 80 was sufficient to cover the surface of nanoscale particles effectively and prevent agglomeration during the homogenization process [24]. Moreover, Polysorbate 80 is classified as low toxicity and is classed as generally recognized as safe (GRAS) among surfactants [11]. For a substance like Polysorbate 80 to be considered GRAS, its safety must be recognized by “experts qualified by scientific training and experience to evaluate its safety,” governed by the US Food and Drug Administration (FDA) [26]. Hence, it is postulated that the addition of Polysorbate 80 would not cause any unwanted side or adverse effects towards the human health.

TQ encapsulation efficiency and drug loading capacity of TQ-NLC were found to be relatively high (97.83 ± 0.1375%). The study suggests that TQ has good solubility in the surfactant (Polysorbate 80) which helps to sustain the compound inside the lipid phase. In addition, the high encapsulation efficiency and drug loading capacity of TQ-NLC may be due to the use of olive oil as one of the components of the lipid matrix as majority of lipophilic compounds including TQ solubilize better in oils [25]. In fact, TQ-NLC formulation provides a weak crystallization as the result of increased imperfection in the crystal lattice. This was due to the binary mixture of liquid (olive oil) and solid lipid (HPO) that provides enough space to accommodate TQ molecules, resulting in higher drug encapsulation efficiency [19, 24, 27]. The high encapsulation efficiency avoids or reduces the wastage of compounds as majority of them are encapsulated inside the nanostructured lipid carrier, hence lowering the production cost.

Differential scanning calorimetry was performed to characterize the polymorphism and the degree of crystallinity of TQ-NLC. The study shows that the melting point of TQ-NLC (56.73°C) was lower than that of the bulk material (HPO) (60.50°C), but higher than TQ (47.35°C), which indicates that TQ was dissolved in the lipid matrix and encapsulated in the nanostructured lipid carriers [25]. During the production, TQ has been dissolved in the melted lipid phase. Following the cooling of the dispersion to room temperature, the melting event of TQ was not detected anymore. The absence of this thermodynamic transition can be due to a molecular dispersed state of TQ in the mixture [25]. The decrease in the melting point of TQ-NLC (56.73°C) and blank NLC (57.18°C), which was below that of bulk materials, HPO (60.50°C), is termed and described as “melting point depression.” This phenomenon indicates that HPO is being transformed into nanoparticulate forms. The melting point depression is attributed to the small diameter of nanoparticles and high specific surface area. The addition of oil (i.e., olive oil) into the matrix provoked an additional shift of the melting point to lower temperature in both TQ-NLC and blank NLC [24, 28]. Decrease in melting enthalpy in NLC and TQ-NLC as compared to HPO and TQ was due to its less-ordered arrangement of nanoscale particles. Hence, lesser amount of energy was needed to overcome the lattice force in the nanoparticles than HPO [29]. In addition, incorporation of TQ inside the lipid matrix results in a further increase in the number of defects in the lipid crystal lattice and hence causes a slightly lower melting point of TQ-NLC (56.73°C) as compared to blank NLC (57.18°C). The defect in the crystalline lattice in TQ-NLC was confirmed by the high drug encapsulation efficiency and drug loading capacity [24].

As shown in Table 3, based on the IC_50_ values, TQ-NLC was most cytotoxic towards MDA-MB-231 compared to HeLa, SiHa, and MCF-7. MCF-7 was found to be less sensitive to TQ-NLC as compared to MDA-MB-231 which is most likely due to the presence of oestrogen receptor in MCF-7 that facilitates cell growth and hampers the induction of apoptosis [30]. TQ-NLC was cytotoxic towards the cells in a time-dependent manner. The IC_50_ values of TQ-NLC towards normal cells (3T3-L1 and Vero) after 72 hours time were significantly higher than those of HeLa and SiHa cells. It shows that TQ-NLC was less cytotoxic towards normal cells. Similar results had been shown by TQ previously. TQ has been reported to show significant cytotoxicity towards HeLa and SiHa cells in a dose- and time-dependent manner. Meanwhile, TQ was less cytotoxic towards the normal cells [4, 5].

In this study, Swiss mouse embryo fibroblast cells (3T3-L1) and African green monkey kidney epithelial (Vero) cells were used to assess the cytotoxicity of TQ-NLC towards normal cells. 3T3-L1 cell line is recommended by US National Institute of Environmental Health Sciences (NIEHS), Interagency Coordinating Committee on the Validation of Alternative Methods (ICCVAM), to access basal cytotoxicity [31]. Vero cells are homologous with human body cells as they share a common embryonic origin (mesoderm) with cells from human genital tract, and this line is nontumorigenic but is immortalized, allowing the culturing of cells for longer than normal cell line [32, 33]. In addition, the rationale behind the use of 3T3-L1 and Vero cells rather than primary cervical breast cancer cells is that these normal cells have been banked and well characterized, thus avoiding the issue of lot-by-lot viability, variations, and adventitious agent contamination of the primary cultures [34].

Low cytotoxicity of blank NLC towards both cancerous and normal cell lines (with percentage viability ranging from 87% to 94%) was noted. It has been reported that the cytotoxicity of blank NLC is due to the usage of Polysorbate 80 rather than solid lipid (HPO) and olive oil [35]. However, as earlier discussed, Polysorbate 80 was introduced in the formulation of TQ-NLC as it acts as a nonionic surfactant which helps to stabilize the nanoformulation [24, 25]. Therefore, the presence of Polysorbate 80 can be considered as an additive value to the TQ-NLC by enhancing the performance of TQ.

MDA-MB-231 cells treated with TQ-NLC exhibited some features of apoptosis such as detachment of cells from the substratum, cells shrinkage, and membrane blebbing as well as formation of apoptotic bodies [36]. The induction of apoptosis was evidenced by the accumulation of cells at sub-G1 phase that indicates the cleavage of nuclear DNA into multiple fragments [37]. Apoptosis form of death is more favourable than necrosis in eliminating cancer cells in that it does not trigger inflammatory response to the neighbouring cells [38]. Therefore, induction of cell apoptosis and targeting the apoptotic pathways have emerged as an attractive approach for treatment of cancer [39]. Many US Food and Drug Administration (FDA) approved anticancer drugs such as paclitaxel (a compound extracted from the Pacific yew tree,* Taxus brevifolia*), camptothecin (an alkaloid isolated from the Chinese tree,* Camptotheca acuminate*), and genistein (soy-derived isoflavone and phytoestrogen) have been found to induce apoptosis [40]. Therefore, the ability of TQ-NLC to induce apoptosis in MDA-MB-231 cells suggests that TQ-NLC may be a potentially effective chemotherapeutic agent against hormonal-independent breast cancer.

Apart from that, TQ-NLC was found to induce non-phase specific cell cycle arrest in MDA-MB-231 cells at different exposure time. TQ-NLC induced cell cycle arrest at G2/M and S phase at 24 and 48 hours. The exact mechanism of the non-phase specific arrest is unclear. The arrest of cell cycle is known to be orchestrated by cyclin-dependent kinases (CDKs). Their activity depends not only on the availability and binding of CDK inhibitors or other regulatory factors but also on phosphorylation/dephosphorylation status of the kinases themselves [41]. The DNA damage-induced cell cycle arrest in the G1 and S phases may partly involve inhibition of the activity of G1 CDKs by the specific CDK inhibitor, p21 [42]. Moreover, the mechanism underlying the DNA damage-induced G2 arrest was shown to involve specific inhibitory phosphorylation of the mitotic kinase, CDK1, in human cells [43, 44]. Some of the examples of non-phase specific clinically available cytotoxic agent include cisplatin (CDDP), 4-hydroperoxy-cyclophosphamide, mitomycin C, and doxorubicin [45]. The cell cycle analysis was not performed on samples of 72 hours of incubation as majority of the cells were not viable. It will be difficult to determine the effect of TQ-NLC on the cell cycle with the presence of a large number of dead cells. Flow cytometry analysis will not be able to determine the cell cycle phase of dead cells as there are no changes in DNA levels to generate characteristic cellular DNA content profiles compared to cells which retain their proliferative ability [46].

## 5. Conclusion

In this study, TQ-NLC which is of nanoscale has been successfully synthesized by the high pressure homogenization method. Even though the surface potential of TQ-NLC was more than −30 mV, it was still stable up to 6 months of storage. Moreover, TQ-NLC also showed high encapsulation efficiency and drug loading capacity. MDA-MB-231 was most sensitive toward TQ-NLC compared to other cancer cell lines (HeLa, SiHa, and MCF-7). Nevertheless, TQ-NLC was relatively noncytotoxic towards normal cells (3T3-L1 and Vero). TQ-NLC induced apoptosis and non-phase specific cell cycle arrest in MDA-MB-231 cells. Thus, TQ-NLC has the potential to be developed into a drug for treatment of breast cancer.

## Figures and Tables

**Table 1:** Physicochemical characteristics of NLCs and TQ-NLCs after synthesis.

Formulation	Duration of storage (week)	Average diameter (nm)	Polydispersity index (PDI)	Zeta potential (mV)
NLC	0	31.25 ± 0.1793^a^	0.161 ± 0.0043^1^	19.68 ± 0.5189^α^
	24	33.11 ± 0.3398^b^	0.172 ± 0.0061^1,2^	16.25 ± 0.7920^β,γ^

TQ-NLC	0	35.66 ± 0.1235^∗c^	0.177 ± 0.0024^∗2^	16.72 ± 0.4474^∗β^
	24	37.05 ± 0.2742^∗d^	0.211 ± 0.0043^∗3^	14.78 ± 0.2470^∗γ^

Values were the means of three replicate samples. The data were presented as mean ± SEM. ∗ were significant as compared to NLC while a, b, c, d, 1, 2, 3, *α*, *β*, and *γ* were significantly different (*P* < 0.05).

**Table 2:** Drug encapsulation efficiency of TQ-NLC at 0 weeks and 24 weeks after synthesis.

Formulation	Duration of storage (week)	Drug encapsulation efficiency (%)	Drug loading capacity (mg per mg of lipid)
TQ-NLC	0	97.63 ± 0.1798	97.63 ± 0.1798
	24	95.94 ± 0.2562^*^	95.94 ± 0.2562^*^

Values were the means of three replicate samples. The data were presented as mean ± SEM. ∗ were significant as compared to 0 weeks of storage duration.

**Table 3:** Cytotoxicity of TQ-NLC as reflected by the IC50 value at various incubation times determined by using MTT assay.

Cell line	IC50 value (*μ*M)		
	24 h	48 h	72 h
Cancerous cell line			
MDA-MB-231	6.50 ± 0.50^a^	4.43 ± 0.12^b^	4.47 ± 0.06^b^
MCF-7	>50	>50	>50
SiHa	19.42 ± 0.33^c^	10.42 ± 0.17^d^	8.50 ± 0.14^e^
HeLa	23.00 ± 0.14^f^	18.17 ± 0.51^g^	15.58 ± 0.17^h^
Normal cell line			
3T3-L1	NP	NP	>50
Vero	NP	NP	32.00 ± 0.29^i^

Values were the means of three replicate samples. The data were presented as mean ± SEM. a, b, c, d, e, f, g, h, and i were significantly different (*P* < 0.05).

NP = not performed.
